# Mastering the allergen risk in chidren nutrition, the Manoe project

**DOI:** 10.1186/2045-7022-1-S1-O5

**Published:** 2011-08-12

**Authors:** Olivier Tranquet, Françoise Le Vacon, François Morgan, Olivier Bonaly, Alain Nouvellon, Freddy Allaire, Mohamed Merdji, Daniel Piveteaul, Martine Drouet

**Affiliations:** 1 INRA French national Institute for Agronomical Research, Nantes, France; 2 Atlangene Silliker, Nantes, France; 3 Lactalis R&D, Retiers, France; 4 SADAC, Maulevrier, France; 5 Charal, Cholet, France; 6 Brioche Pasquier, Cerqueux, France; 7 Audencia, Nantes, France; 8 AFPRAL, Saint Michel sur Orge, France; 9 CHU Angers, Angers, France

## 

Regulatory agencies have enforced allergen labelling rules for food ingredients on pre-packed food. HACCP procedures driven on by food manufacturers reveals that possible cross-contaminations of their products cannot be absolutely discarded or checked. As a result and because allergen absence cannot be accurately warranted in most food industry, numerous warning mentions on pre-packed foods appeared to inform allergic consumers of potential cross contaminations even if levels are very low. For Allergic consumers, accessibility to food is clearly restricted.

In a practical view, the Unit of Allergology in Angers Hospital (France) has developed an oral reintroduction protocol of low doses of allergens to evaluate the sensitivity of allergic patients. It appeared that most of the patients tolerate low amount (few mg) of allergen and would support the consumption of product contaminated by traces of allergen.

Based on these facts, the French competitiveness cluster "Pôle Enfant" gathered four food manufacturers (pastry, meat, milk products: BRIOCHE PASQUIER, CHARAL, LACTALIS, SADAC), ATLANGENE®-SILLIKER specialized in food analysis, ten university hospitals and hospitals, 3 academic laboratories (INRA, CNRS, and AUDENCIA), and an association of French allergic consumers (AFPRAL) to construct the "MANOE project". This project funded by the regional council of Pays de la Loire aims to develop food products designed for the general population which would be tolerated by children allergic to peanut, egg, milk or wheat. In a large multicentric clinical trial, 400 allergic children will be submitted to the standardized reintroduction protocol. The acceptance and the usefulness of these new products by allergic children and their parents will be evaluated.

In the context where thresholds values for allergen labelling rules are in discussion all around the world, this integrated project will provide a practical insight on how food companies will deal with thresholds, if the analytical methods are good enough to guaranty these levels, and how the patient will use this new information.

